# Open or robotic? Radical cystectomies for patients with non-metastatic bladder cancer: A systematic review and meta-analysis

**DOI:** 10.1017/cts.2024.493

**Published:** 2024-03-15

**Authors:** Jada Ohene-Agyei, Marisha Madhira, Holly Smith, Mihaela E. Sardiu, Eugene K. Lee

**Affiliations:** 1 University of Missouri-Kansas City, Kansas City, MO, USA; 2 University of Kansas Medical Center, Kansas City, KS, USA

**Keywords:** Bladder cancer, urology, surgical modalities, robotic surgery, da vinci

## Abstract

**Background:**

This systematic review and meta-analysis will review randomized control trials for localized bladder cancer, evaluating surgical and pathologic outcomes of ORC versus RARC.

**Methods:**

Randomized studies evaluating adults with non-metastatic bladder cancer who underwent a radical cystectomy. Randomized trials were selected for final review. Data was extracted and analyzed with Revman 5 software. The primary outcome was complication rates within 90 days. Secondary outcomes included postoperative quality of life, estimated intraoperative blood loss, and other perioperative outcomes. Continuous variables were reported using mean difference with 95% confidence intervals, and dichotomous variables were reported using risk difference with 95% confidence intervals with RARC as the experimental group and ORC as the reference group.

**Results:**

Of 134 articles screened, six unique randomized studies were selected. For Grade I-II complications, the risk ratio (RR) was 0.92 (95% CI [0.79,1.08], *p* = 0.33), and for Grade III-V complications, RR 0.93 (95% CI [0.73,1.18], *p* = 0.59). RARC resulted in decreased blood loss (95% CI [−438.08, −158.44], *p* < 0.00001) and longer operative time (95% CI [55.23, 133.13], *p* < 0.00001). Quality of life using the EORTC-QLQ-30 global health score at 3 months post-op appeared to favor RARC with a mean difference of 4.46 points (95% CI [1.78, 7.15], *p* = 0.001). Pathologic outcomes neither statistically nor clinically favored one modality, as there was no significant difference between mean lymph node yield (*p* = 0.49), positive lymph nodes (*p* = 1.00), and positive surgical margins (*p* = 0.85) between the surgical modalities.

**Conclusions:**

Although one surgical modality is not overtly superior, the choice may be decided by mitigating individual operative risk factors like intraoperative blood loss, operative time, post-operative quality of life, as well as institutional costs and learning curve among surgeons.

## Introduction

Bladder cancer (BC) is the most common cancer of the urinary tract, with higher prevalence in Western nations due to increased exposure to carcinogens. It is the 10^th^ most diagnosed cancer, accounting for around 80,000 new cases and 17,000 deaths in the United States annually. Risk factors include advanced age and exposure to carcinogens such as tobacco smoke (and less commonly benzene compounds and aromatic amines). The lifetime risk of BC is about 1.1% for males and 0.27% for females [1]. Though non-Hispanic White males have a higher incidence of BC, female patients tend to have poorer outcomes, as the condition typically is more advanced at time of diagnosis [2].

BC presents as either non-muscle-invasive bladder cancer (NMIBC), muscle-invasive bladder cancer (MIBC), or metastatic disease. Prognosis worsens significantly upon invasion of muscularis propria [3,4]. Radical cystectomy with bilateral lymph node dissection is the mainstay surgical treatment for patients with high-risk NMIBC (that is, recurrent or persistent carcinoma after intravesical Bacillus Calmette–Guérin therapy and MIBC [5].

First described in the 1940s by Marshall and Whitmore, the radical cystectomy remains one of the most technically difficult and morbid surgical procedures in urology [5]. Historically, it is a way of resecting the tumor by “removing the bladder along with the prostate and seminal vesicles in men, and uterus, fallopian tubes, ovaries, and anterior vagina in women.” It is often followed by lymph node dissection, which provides important prognostic information, and urinary diversion [5,6]. An open radical cystectomy (ORC) through a lower midline incision was the typical approach; however, in recent years, the robot-assisted radical cystectomy (RARC) has increased in the United States [7]. While RARC has been shown to be expensive, it has demonstrated the equal treatment of cancer with shorter hospital stays and fewer perioperative transfusions [8]. In this systematic review and meta-analysis, we will review randomized control trials assessing the perioperative and postoperative outcomes of ORC and RARC for bladder cancer patients, excluding those with metastatic disease.

A previous systematic review and meta-analysis by Sathianathen et al found no considerable difference in oncological, safety, and quality of life outcomes’ with RARC versus ORC [9]; however, more recent randomized trials have been published since the time of Sathianathen’s work in 2018. Recent literature suggests improvements in days alive and out of the hospital with RARC compared to ORC, and nonsignificant differences in secondary outcomes like quality of life, complications, and activity levels [10]. Further analysis is required to continue to inform clinical guidelines with the evidence provided by previous and recent randomized controlled trials.

## Objective

The objective of this systematic review is to compare the primary outcome of 90-day complication rates, as well as the secondary perioperative and pathologic outcomes and quality of life between non-metastatic bladder cancer patients who received an RARC versus ORC. The lack of quantitative data from previous studies evaluating similar outcomes has been a limitation in the current body of evidence, thus prompting the authors to include a meta-analysis.

## Methods

### Eligibility criteria

Our patient population included studies comparing adults (aged ≥18) with non-metastatic bladder cancer who underwent a radical cystectomy via RARC as the intervention and ORC as the control. This review included all urinary diversion techniques. Studies including patients with metastatic disease were excluded from this review. Additionally, adults undergoing radical cystectomy for purposes other than bladder cancer, patients who have undergone previous abdominal/pelvic surgeries or radiation, and patients with anesthetic contraindications to major pelvic surgery were excluded from this review if these patients were explicitly included as the study population in the studies included Non-randomized studies were excluded. Included articles were no older than 10 years (2012–2022) and published in journals internationally.

### Information sources

Studies were selected from PubMed, Embase, and Web of Science between September 2022 and October 2022; the date of the last search was October 21st, 2022.

### Search strategy

Review authors searched for trials with the keywords “radical cystectomy,” “robot-assisted” or “robotic” or “robot-assisted surgery,” and “open” in the title/abstract engine. Then, in all fields, review authors searched “postoperative complications” and “quality of life” or “health related quality of life.” We placed filters restricting to only studies published in the last 10 years (January 1, 2012, to October 21st, 2022), prospective clinical trials, and randomized. Combined, we yielded 14 studies from PubMed, 74 studies from Embase, and 46 studies from Web of Science (see Appendix A).

### Selection process

**Figure 1.**
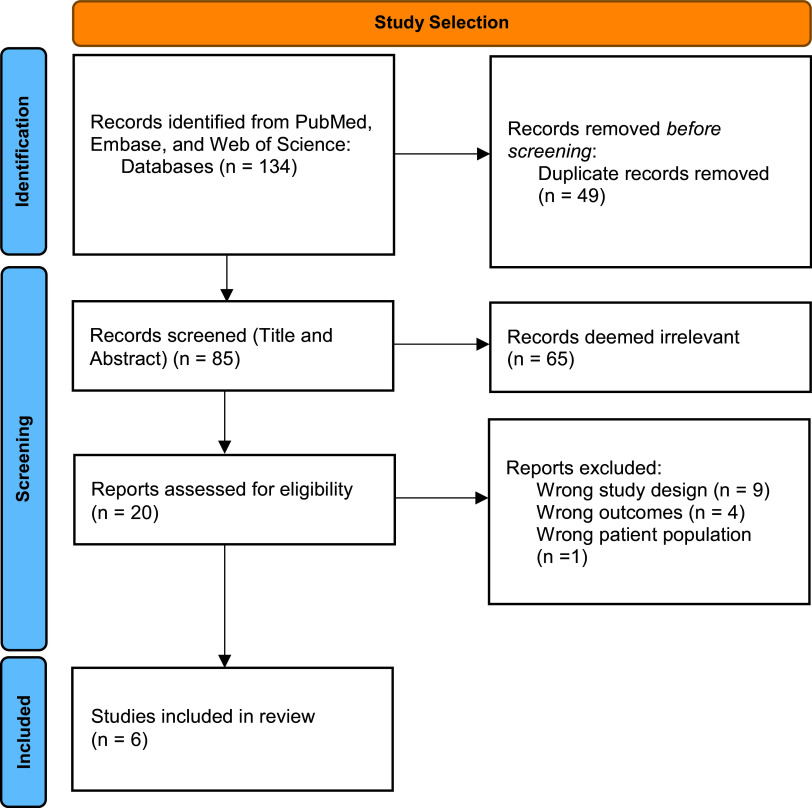
Flow diagram of study selection. Organized with the assistance of Covidence®.

One hundred thirty-four study records from the three databases were imported into Covidence for duplicate removal and screening. Articles were selected based on the study design, outcomes evaluated, and study population (see Fig. 1). Selected studies included randomized clinical trials that evaluated postoperative patient outcomes (excluding cost analysis as the primary outcome) and quality of life, not limited to a particular subgroup of adults with bladder cancer. Publications regarding the same trial but with non-overlapping outcomes were included in the study, assuming they fulfilled the other inclusion criteria. All data were collected from either the manuscript or supplementary materials from each article. Information from individual correspondence from authors was not included. Six unique randomized controlled trials were included in this review, three parent trials with subsequent studies publishing unique or long-term data (see Table 1). MM and JO equally and individually screened and reviewed articles. If discrepancies could not be resolved via discussion, they were resolved by the senior author (EL).

**Table 1.** Summary of selected studies

Reference	Year of Publication	Clinical Trial Country	Methodology	Primary Aim	Outcomes
Bochner *et al*. [11]	2015	United Stated of America	Two-arm RCT where patients were randomized to either ORC/PLND^a^ or RARC/PLND, both with open urinary diversion.	To compare perioperative complications between RARC and ORC techniques	90-day complication rate (primary), EBL, operative time, pathologic outcomes, 3- and 6-month QoL, total operating room, and inpatient costs (secondary).
Bochner *et al*. [12]	2018	United Stated of America	Analysis of oncologic outcomes of surviving patients for up to two years.	To compare cancer outcomes in BC patients managed with ORC or RARC.	Two -year recurrence-free, cancer-specific, and overall survival.
Catto *et al*. [13]	2022	United Kingdom	Multicenter two-arm RCT where patients were randomized to either RARC with intracorporeal urinary diversion or ORC.	To compare recovery and morbidity after RARC with intracorporeal reconstruction vs. ORC	Number of days alive and out of the hospital within 90 days of surgery (primary), and oncologic outcomes, quality of life, disability, stamina, activity levels, and survival (secondary).
Khan *et al*. [14]	2016	United Kingdom	Three-arm single-center RCT where patients were randomized to ORC, RARC, or laparoscopic radical cystectomy.	To compare the postoperative outcomes of patients undergoing ORC, RARC, and laparoscopic radical cystectomy.	30-day and 90-day complication rates (primary), operative time, EBL, delay in bowel function, LOS, margin status and a number of lymph nodes retrieved, 12-month oncological outcomes, QoL (secondary).
Khan *et al*. [15]	2020	United Kingdom	Analysis of prospectively maintained database curated during and after parent trial.	To evaluate the 5-yr oncological outcomes of patients recruited into the parent trial.	The outcomes of interest included 5-yr recurrence-free survival, cancer-specific survival, and overall survival.
Maibom *et al*. [16]	2022	Denmark	Single center, two-arm feasibility study where patients were randomized to ORC or RARC with intracorporeal diversion.	To examine surgical outcomes and the feasibility of blinding patients and care providers to the surgical technique of radical cystectomy.	Proportion of unblinded patients and success of blinding using Bang’s Blinding Index (primary), LOS, complication rates, EBL, pain, and opioid consumption (secondary).
Vejlgaard et al. [17]	2022	Denmark	Predefined secondary analysis of a single-center, double-blinded, randomized feasibility trial.	To examine the QoL before and after radical cystectomy RC and compare RARC to ORC.	Patient-reported QoL using the EORTC Cancer-30 and muscle invasive bladder cancer BLM-30 QoL questionnaires before and after RC.
Mastroianni *et al*. [18]	2022	Italy	Single-center, two-arm RCT where patients were either randomized to ORC or RARC with intracorporeal urinary diversion.	To report an interim analysis of 1-yr health-related QoL outcomes from an ongoing randomized controlled trial comparing ORC and RARC with totally intracorporeal urinary diversion.	Demonstration of RARC superiority with 50% reduction in transfusion rate compared to the ORC group (primary), LOS, 30-, 90-, and 180-day complication rates, global costs analysis, 6-month functional, oncologic and HRQoL outcomes, continence based on a number of pads used per day (secondary).
Parekh *et al*. [19]	2018	United States of America	Multicenter, two-arm, randomized, open-label, non-inferiority, phase 3 trial where patients were randomized to RARC or ORC with extracorporeal urinary diversion.	The Randomised Open versus Robotic Cystectomy (RAZOR) trial was designed to investigate whether robot-assisted radical cystectomy was non-inferior to open radical cystectomy for treating bladder cancer.	The primary endpoint was progression-free survival at two years after surgery. The secondary endpoints were blood loss, the proportion of patients requiring blood transfusion, surgical margin status, number of lymph nodes resected, operating time, length of hospital stay, surgical complications at 90 days, and change in health-related QoL outcomes at 3 and 6 months.

ORC = open radical cystectomy; RARC = robot-assisted radical cystectomy; PLND = pelvic lymph node dissection; EBL = estimated blood loss; LOS = length of stray; QoL = quality of life; HRQoL = health-related quality of life.

Of note, this systematic review and meta-analysis includes data from six unique trials, but as shown in the Table above, data from certain trails were published in separate manuscripts. Alternating colors delineate one unique trial from another.

### Data collection process

The data from variables of interest were extracted equally and individually by MM and JO and entered into the Cochrane Review Manager 5.4.1(RevMan) software. Continuous variables were entered into the software as mean and standard deviation. Dichotomous variables were entered as *n* number of occurrences per study group.

### Data items

The following baseline characteristics from the population used in each study were collected: age, number of males, percentage of patients who received an ileal conduit, pathological stage of T2 or higher, and percentage of patients who received neoadjuvant chemotherapy. Not all studies included ethnic/racial demographic information, and thus this was not included as part of the collected baseline characteristics. The primary outcome of interest was a 90-day complication rate sub-grouped by the Clavien-Dindo grading scale. Secondary outcomes of interest included operation time, estimated intraoperative blood loss, length of stay, lymph node yield, number of patients with positive lymph nodes, positive surgical margins, and quality of life. Quality of life was assessed using the European Organization for Research and Treatment of Cancer (EORTC) QLQ-C30 questionnaire, a health-related quality of life questionnaire designed specifically for cancer patients [20]. Study outcomes were compatible with the aforementioned domains.

### Study risk of bias assessment

The Cochrane Risk of Bias 2 (RoB2) Tool was used to assess the risk of bias in all included studies. RoB2 consists of five domains: randomization process, deviations from the intended interventions, missing outcome data, measurement of the outcome, and selection of the reported result. Each domain was then evaluated for the overall risk of bias assessment. ROBINS-I tool was used to assess the risk of bias in non-randomized studies. MM and JO individually determined the risk of bias in each study and resolved discrepancies via discussion.

### Effect measures

In accordance with the Cochrane Handbook for Systematic Reviews of Interventions, continuous outcomes were analyzed with the inverse variance method using a random effects model and reported as the mean difference with 95% confidence intervals. Dichotomous outcomes were analyzed with the Mantel-Haenszel statistical method using a random effects model and reported as risk ratios with a 95% confidence interval. All statistical analyses were calculated in RevMan.

### Synthesis methods

Each study collected participants’ baseline characteristics and surgical outcomes, comparing the RARC to the ORC. Khan et al. 2016 did have an additional laparoscopic radical cystectomy arm, not included in this review. There was varied reporting of continuous outcomes among the studies, in that some studies reported outcomes as mean and standard deviation, and others reported as median and interquartile range. Continuous variables reported in the median and interquartile range were approximated to their respective mean and standardized deviation by assuming the median to represent the mean, and interquartile range was approximated to the standard deviation using the approximation equation provided by Cochrane. Heterogeneity was assessed using the Chi^2^ statistic using *n*-1 degrees of freedom and the I^2^ statistic.

### Reporting bias assessment

Protocols from each included study were examined; this review did not include studies without a previously published protocol, or pre-specified outcomes that drastically deviated from the published trial studies.

### Certainty assessment

Certainty of evidence was determined individually by JO and MM using the Grading of Recommendations, Assessment, Development, and Evaluation (GRADE) Pro software. Discrepancies were resolved via partner discussion. For each outcome, the certainty assessment considered the number of studies, study design, overall risk of bias, inconsistency of results across studies, indirectness of results, imprecision, and other considerations. Then, using the total number of participants from whom data was extracted and the appropriate effect measures, a summary of findings was generated to grade the available evidence on a high, moderate, low, and very low scale (see Table 2).

**Table 2.** GRADE pro certainty of evidence determination for each outcome

Outcomes	№ of participants (studies) Follow-up	Certainty of the evidence (GRADE)	Relative effect (95% CI)	Risk difference with RARC
90d Complications - Grade I-II	835 (5 RCTs)	⨁⨁⨁⨁ High^a^	**RR 0.92** (0.79 to 1.08)	**37 fewer per 1,000** (97 fewer to 37 more)
90d Complications - Grade III-V	953 (6 RCTs)	⨁⨁⨁⨁ High^a^	**RR 0.93** (0.73 to 1.19)	**15 fewer per 1,000** (57 fewer to 40 more)
Operative Time (OT) assessed with: minutes	953 (6 RCTs)	⨁⨁⨁◯ Moderate^a^	–	MD **94.19 mL more** (55.25 more to 133.13 more)
Length of Stay (LOS) assessed with: days	953 (6 RCTs)	⨁⨁⨁◯ Moderate^a^	–	MD **0.19 days lower** (1.15 lower to 0.77 higher)
Estimated Intraoperative Blood Loss (EBL) assessed with: mL	953 (6 RCTs)	⨁⨁⨁◯ Moderate^a^	–	MD **308.39 mL fewer** (458.34 fewer to 158.44 fewer)
Positive Lymph Nodes assessed with: no. of patients	475 (3 RCTs)	⨁⨁⨁⨁ High	**RR 1.00** (0.66 to 1.50)	**0 fewer per 1,000** (55 fewer to 81 more)
Positive Surgical Margins assessed with: no. of Patients	953 (6 RCTs)	⨁⨁⨁⨁ High	**RR 1.06** (0.60 to 1.88)	**3 more per 1,000** (20 fewer to 45 more)
Lymph Node Yield assessed with: no. of lymph nodes	953 (6 RCTs)	⨁⨁⨁◯ Moderate^a^		MD **0.59 lymph nodes lower** (2.27 lower to 1.09 higher)
Quality of Life at 3 months (QoL-3) assessed with: EORTC QLQ-C30 follow-up: mean 3 months	485 (3 RCTs)	⨁⨁⨁◯ Moderate^a^	–	MD **4.46 points higher** (1.78 higher to 7.15 higher)
Quality of Life at 6 months (QoL-6) assessed with: EORTC QLQ-C30 follow-up: mean 6 months	551 (3 RCTs)	⨁⨁⨁◯ Moderate^a^		MD **0.66 points higher** (2.45 lower to 3.78 higher)

ORC = open radical cystectomy; RARC = robot-assisted radical cystectomy; QoL = quality of life; RCT = randomized control trial; GRADE = Grading of Recommendations, Assessment, Development, and Evaluation; EORTC QLQ-C30 = quality of life assessment tool; CI = confidence interval; MD = mean difference; RR = risk ratio.

The risk in the intervention group (and its 95% confidence interval) is based on the assumed risk in the comparison group and the relative effect of the intervention (and its 95% CI).

**GRADE Working Group Grades of Evidence**

**High certainty**: We are very confident that the true effect lies close to that of the estimate of the effect.

**Moderate certainty**: We are moderately confident in the effect estimate: the true effect is likely to be close to the estimate of the effect, but there is a possibility that it is substantially different.

**Low certainty**: Our confidence in the effect estimate is limited: the true effect may be substantially different from the estimate of the effect.

**Very low certainty**: We have very little confidence in the effect estimate: the true effect is likely to be substantially different from the estimate of effect.

**Explanations**

a Differences in reporting of continuous surgical outcomes (mean and standard deviation vs. median and interquartile range).

## Results

### Results synthesis

One hundred thirty-four study records from the three databases were imported into Covidence for duplicate removal and screening. Six unique randomized trials were included in this review (see Table 3). We were unable to perform subgroup analysis due to insufficient data.

**Table 3.** Summary of participant baseline characteristics

Study	RARC						ORC					
	No. patients	Age	Males	Received Ileal Conduit	pT2 or Greater	Received Neoadjuvant Therapy	No. patients	Age	Males	Received Ileal Conduit	pT2 or Greater	Received Neoadjuvant Therapy
Bochner et al. 2015 [11]	60	66 (8.2)	51 (85.0)	27 (45.0)	25 (41.7)	19 (31.7)	58	65 (8.2)	42 (72.4)	23 (39.7)	26 (44.8)	26 (44.8)
Catto et al. 2022 [13]	161	69.3 (8)	80 (49.7)	88 (54.7)	50 (31.0)	54 (33.5)	158	68.7 (8.4)	78 (49.4)	90 (57.0)	50 (31.6)	53 (33.5)
Khan et al. 2016 [14]	20	68.6 (6.8)	17 (85.0)	18 (90.0)	9 (45.0)	2 (10.0)	20	66.6 (8.8)	18 (90.0)	17 (85.0)	6 (30.0)	3 (15.0)
Maibom et al 2022 [16]	25	70 (8.2)	18 (72.0)	19 (76.0)	16 (64.0)	9 (36.0)	25	67 (11.1)	20 (80.0)	18 (72.0)	19 (76.0)	10 (40.0)
Mastroianni et al 2022 [18]	58	64 (12.6)	44 (75.9)	–	30 (51.7)	23 (39.7)	58	66 (9.6)	40 (69.0)	–	28 (48.3)	22 (37.9)
Parekh et al 2018 [19]	159	70 (34.8)	126 (79.2)	113 (71.1)	84 (52.8)	41 (25.8)	153	67 (35.5)	128 (83.7)	122 (79.7)	82 (53.6)	55 (35.9)

Reported as n (%), with the exception of “Age,” which was reported as mean (SD).

pT2 = Pathologic Stage T2; ORC = open radical cystectomy; RARC = robot-assisted radical cystectomy.

### Primary outcome

For the primary outcome of postoperative complication rates up to 90 days, a nonsignificant difference in rates of 90-day complications for all grades was found between the RARC and ORC groups. For the occurrence of Grade I-II complications, the risk ratio (with ORC as the reference group) was 0.92 (95% CI [0.79,1.08], *p* = 0.33), and for Grade III-V complications, RR 0.93 (95% CI [0.73,1.18], *p* = 0.59). The certainty of evidence for these findings was high.

### Secondary outcomes

#### Estimated intraoperative blood loss

All six studies reported data on the estimated blood loss for each participant in their trial, with 483 patients for the RARC group and 470 for the ORC group. It was found that performing a cystectomy robotically results in decreased blood loss, with a mean difference of −332.8 mL (95% CI [−455.64, −209.97], *p* < 0.00001) with moderate certainty of evidence.

#### Operative time

All six studies reported data on operative time for each trial participant, with 683 patients for the RARC group and 658 for the ORC group. A robotic radical cystectomy was found to take longer, with a mean difference of 94.19 minutes (95% CI [55.25, 133.13], *p* < 0.00001), with a moderate certainty of evidence.

#### Length of stay

Whether a radical cystectomy was performed robotically or open did not seem to affect a patient’s postoperative length of hospital stay significantly; the mean difference was −0.19 days between the RARC and ORC group (95% CI [−1.15, 0.77], *p* = 0.70).

#### Quality of life

Three studies evaluated a baseline and postoperative QoL assessment using the EORTC QLQ-C30 questionnaire, with 279 patients in the RARC group and 272 in the ORC group. At baseline, the two groups did not appear to have significant variations in quality-of-life assessments (mean difference was 0.33 95% CI [−1.93, 2.58], *p* = 0.78. At three months post-cystectomy, a significant difference favoring the RARC patients was present with a mean difference of 4.46 points (95% CI [1.78, 7.15], *p* = 0.001), with moderate certainty of evidence. A 6-month postoperative time point was also collected, only by two studies, but with nonsignificant findings (see Table 4).

**Table 4.** Primary and secondary outcomes summary table

Outcome	Included Studies	Risk Ratio/ Mean difference	95% CI	p-value	Certainty of Evidence	Conclusion
Grade I and II 90d Complication Occurrence	Catto 2022 [13] Khan 2016 [14] Maibom 2022 [16] Mastroianni 2022 [18] Parekh 2018 [19]	0.92	[0.79, 1.08]	0.33	High	No significant difference in 90d Grade 1-II complication occurrence between Orc and RARC.
Grade III - V 90d Complication Occurrence	Bochner 2015 [11] Catto 2022 [13] Khan 2016 [14] Maibom 2022 [16] Mastroianni 2022 [18] Parekh 2018 [19]	0.93	[0.73, 1.19]	0.57	High	No significant difference in 90d Grade III-V complication occurrence between Orc and RARC.
Estimated Intraoperative Blood Loss	Bochner 2015 [11] Catto 2022 [13] Khan 2016 [14] Maibom 2022 [16] Mastroianni 2022 [18] Parekh 2018 [19]	−308.39	[−458.34, −158.44]	<0.0001	Moderate	RARC results in about 308.39 mL less blood loss compared to ORC
Operative Time	Bochner 2015 [11] Catto 2022 [13] Khan 2016 [14] Maibom 2022 [16] Mastroianni 2022 [18] Parekh 2018 [19]	94.19	[55.25, 133.13]	<0.0001	Moderate	RARC results in 94.19 minutes more operative time compared to ORC
Length of Stay	Bochner 2015 [11] Catto 2022 [13] Khan 2016 [14] Maibom 2022 [16] Mastroianni 2022 [18] Parekh 2018 [19]	−0.19	[−1.15, 0.77]	0.70	Moderate	No significant difference in hospital length of stay between RARC and ORC.
3-month Quality of Life	Bochner 2015 [11] Catto 2022 [13] Vejlgaard 2022 [17]	4.46	[1.78, 7.15]	0.001	Moderate	There is moderate evidence that RARC improves 3-month QoL postoperatively
6-month Quality of Life	Bochner 2015 [11] Catto 2022 [13] Mastroianni 2022 [18]	0.66	[−2.45, 3.78]	0.68	Moderate	There is moderate evidence that no significant difference exists between 6-month post-op QoL between RARC and ORC.
Lymph Node Yield	Bochner 2015 [11] Catto 2022 13 Khan 2016 [14] Maibom 2022 [16] Mastroianni 2022 [18] Parekh 2018 [19]	−0.59	[−2.27, 1.09]	0.49	Moderate	There is moderate evidence that there is no significant difference in lymph node yield between RARC and ORC.
No. Patients with Positive Lymph Nodes	Bochner 2015 [11] Catto 2022 [13] Khan 2016 [14]	1.00	[0.66, 1.50]	0.99	High	There is strong evidence that no significant difference in positive lymph node occurrence exists in patients who receive RARC compared to ORC.
No. Patients with Positive Surgical Margins	Bochner 2015 [11] Catto 2022 [13] Khan 2016 [14] Maibom 2022 [16] Mastroianni 2022 [18] Parekh 2018 [19]	1.06	[0.60, 1.88]	0.85	High	There is strong evidence that no significant difference in positive surgical margin occurrence exists in patients who receive RARC compared to ORC.

Risk ratio and mean difference with 95% confidence intervals were determined using RevMan® software.

ORC = open radical cystectomy; RARC = robot-assisted radical cystectomy; QoL = quality of life.

#### Lymph nodes

All six studies reported an estimate of their lymph node yield for each cohort. There was no difference in the average lymph node yield between the RARC and ORC groups (mean difference found to be −0.59 95% CI [−2.27, 1.09], *p* = 0.49). The same was found for the incidence of positive lymph nodes in found cystectomy; however, only half of the studies reported this information. The risk ratio was found to be 1.00 (95% CI [0.66, 1.51], *p* = 1.00).

#### Surgical margins

All six studies reported their incidence of positive surgical margins for each patient cohort. No significant difference in positive surgical margins between the RARC and ORC groups was found. The risk ratio of the positive surgical margins found was 1.06 (95% CI [0.60, 1.88], *p* = 0.85).

#### Risk of bias

**Figure 2.**
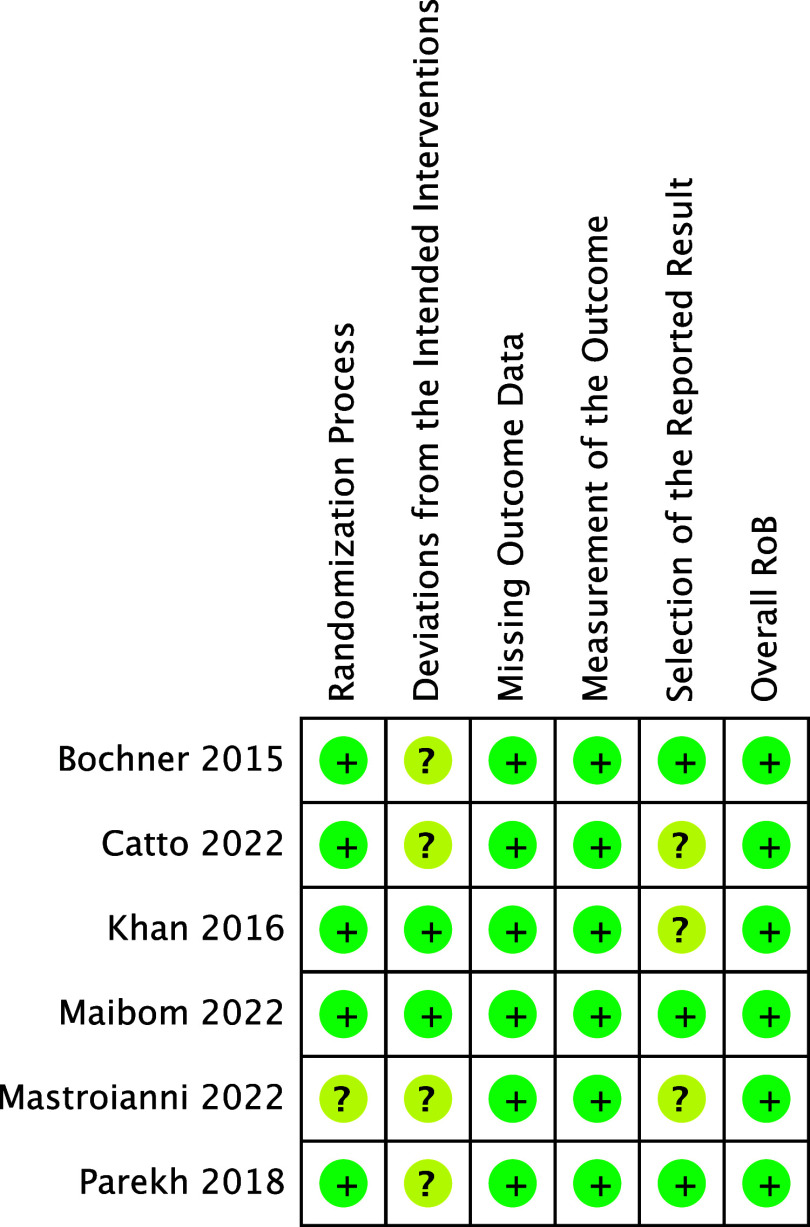
Risk of bias table. Determined using RevMan® software.

All included studies were designated to have an overall low risk of bias. Several studies had concerns in the “Deviations from Intended Interventions” ROB2 domain because the studies were not blinded, and thus, surgery type was sometimes, changed. These studies completed an appropriate intent-to-treat analysis and were used per protocol. Inconsistency arose where individual outcomes (ex: QoL endpoints) were reported per protocol instead of intent-to-treat (see Fig. 2).

#### Heterogeneity and sensitivity analysis

Differences in surgery personnel, personnel experience, technique, and equipment could contribute to heterogeneity in the collected data. Sensitivity analysis of length of stay, operative time, and estimated intraoperative blood loss for Bochner et al. and Khan et al. (Appendix C) to analyze differences in effect from the conversion of reported median and interquartile range to mean and standard deviation. Analysis revealed that the direction effect was not changed between the overall group and sensitivity analysis group; however, the latter effect had widened confidence intervals and heterogeneity. This is likely partly due to the difference in using six studies compared to three for the sensitivity analysis. We accounted for this in the certainty of evidence assessment.

## Discussion

Urologists have pioneered innovative surgical technology for decades. They were the first surgeons to incorporate laparoscopy and laser into common procedures. The robotic radical cystectomy was first performed in 2002 using the original da Vinci robotic system [21]. As technology has advanced throughout the years, most urologists strongly support the utilization of robotics in major surgeries [22].

Institutional cost and personnel considerations play a role in the modality of surgery a patient receives. Depending on the caseload of both the surgeons and the hospital itself, a robotic cystectomy costs up to $4000 more than an open cystectomy due to equipment and physician costs; however, prior single-centered studies have demonstrated that these costs are often offset by decreased inpatient length of stay and costs for medications, transfusions, complication treatments, and readmissions within 30 days [23]. A prospective randomized control trial evaluating the cost difference between the two modalities among experienced surgeons is necessary to identify a true cost difference [24].

The learning curve for incorporating robotic surgery into surgical training is complex. Caseload, institution size, procedure type, and several other variables play a role in a trainee’s time to proficiency in robotic surgery. These variables are also confounding and make it difficult to determine the optimal circumstances for curating robotic skills when comparing studies. Pernar et al. conducted a systematic review in 2017 investigating the methods used to define and measure the learning curves for performing robotic surgery. The review experienced many limitations as methods for training surgeons were ill-defined, and there was significant variability in the performance threshold used to determine competency. It was found that a trainee needed to perform between 12 and 140 cases for urologic procedures, with the most common metric for determining competency being total operation time [25]. Nevertheless, robotic training programs are becoming more common, and several small studies have demonstrated that participation in a training program appears to decrease the time to overcome the learning curve.

The evidence suggests that no significant difference in the surgical modalities exists for the primary outcome of overall 90-day complications. However, it is also to be noted that complications were reported differently among studies. The type of surgery also is unlikely to affect overall complications stratified by type (see Appendix B). As demonstrated in this review, the most common postoperative complication of the gastrointestinal variety. Some have speculated that excess manipulation of the bowel during the reconstructive portion of the surgery contributes to the incidence of such complications and therefore advocate for the intracorporeal approach. Most radical cystectomies are accompanied by extracorporeal diversion, most notably the ileal conduit; however, current literature does not suggest a significant difference in gastrointestinal complications between the two [23–25].

The effect of surgery type is unlikely to make a meaningful difference in length of stay, with a mean difference of 0.19 days. Considering clinical outliers that require an unusually long hospital course, the median is arguably the superior measure of central tendency for the LOS outcome, as it is not as susceptible to change with radical data points. Thus, a potential difference in length of stay should not play into the decision on whether to proceed with a robot-assisted or open approach for the radical cystectomy.

We compared our results to a meta-analysis by Khetrapal et. al, which analyses eight meta-analyses; however, upon further review, two of the manuscripts analyzed were from the same clinical trial. We also excluded one study (Nix et. al) that Khetrapal included due to its date of publication in 2010. Khetrapal et, al also report a similar LOS mean difference (0.21 reported by Khetrapal and −0.19 by our group) which was stated as statistically significant by Khetrapal. It is encouraging that our methods yielded similar results. Even with a statistically significant result, 0.2-day difference is not clinically meaningful [26].

There is strong evidence to support that the mean operative time of RARC is greater than ORC; however, several factors play a role in the heterogeneity in operative time data. First, a surgeon’s familiarity and experience with robotic surgery influence the operative time, making comparative studies assessing operative time difficult [27]. Secondly, the included studies did not specify their definition of the operation start time, that is, whether they considered set-up time or not. Thirdly, logistical, staffing, and case variability impact operating room efficiency. Nonetheless, most of the literature would suggest that a robotic case warrants a longer operative time than the open approach.

Limitations of data analysis include the conversion of medians and interquartile ranges to mean and standard deviation, as well as any assumptions made during data extraction and analysis. Although previous sensitivity analysis did not show a difference in effect direction (if it had one), it does impact the mean difference’s true value, which could impact clinical interpretation. Furthermore, the lack of racial and ethnic distribution data impacts the generalizability of our results. However, other factors such as the age and clinical features of the included patients may be generalized to other patients with bladder cancer patients, as 80% of patients diagnosed with bladder cancer are age 65 and older, and about 30% of newly diagnosed bladder cancer is found to be muscle-invasive (pathologic stage T2 and greater). Finally, the inclusion of international studies may mask healthcare issues unique to a specific country, such as the role of health insurance in the United States in helping to determine the most appropriate treatment modality; however, information such as this was not published by the trials nor collected by our team.

## Conclusions

The current evidence shows choice of RARC versus ORC has indiscriminate differences in post-operative complications and quality of life outcomes. Assuming that these outcomes are truly equal, the choice of surgery may be decided by mitigating operative risk factors like blood loss and operative time or cost and learning curve.

## Supporting information

Ohene-Agyei et al. supplementary material Ohene-Agyei et al. supplementary material

## Supplementary material

The supplementary material for this article can be found at https://doi.org/10.1017/cts.2024.493.

## Other information

Registration: PROSPERO ID CRD42022370742.
