# Supplementary material for: Open or robotic? Radical cystectomies for patients with non-metastatic bladder cancer: A systematic review and meta-analysis
Source: J Clin Transl Sci. 2024 Mar 15;8(1):e57. doi: 10.1017/cts.2024.493 (PMC11036446; doi:10.1017/cts.2024.493)
Supplement: Ohene-Agyei et al. supplementary material [file S205986612400493Xsup001.docx]

**Appendix A**

#1 "radical cystectomy"[Title/Abstract]

#2 "robot-assisted"[Title/Abstract] OR "robotic"[Title/Abstract] OR “robot assisted surgery” [Title/Abstract]) AND

#3 "open"[Title/Abstract] AND

#4"postoperative complications"[All Fields]

#5 ("quality of life"[Title/Abstract] OR "health related quality of life"[Title/Abstract])

#6 #1 AND #2 AND #3 AND (#4 OR #5)

Filters: Studies from Jan 01 2012 – October 01 2022

Study types: Clinical Trials, prospective, randomized

Filter Specifics

**PubMed**: “Filters: Clinical Trial, Randomized Controlled Trial, from 2012/1/1 - 2022/10/1”

**Embase**: “AND (**2013**:py OR **2014**:py OR **2015**:py OR **2016**:py OR **2017**:py OR **2018**:py OR **2019**:py OR **2020**:py OR **2021**:py OR **2022**:py) AND (**'clinical study'**/de OR **'controlled study'**/de OR **'major clinical study'**/de OR **'multicenter study topic'**/de OR **'phase 3 clinical trial'**/de OR **'phase 3 clinical trial topic'**/de OR **'prospective study'**/de OR **'randomized controlled trial'**/de OR **'randomized controlled trial topic'**/de)”

**Web of Science:** Publication Years 2022 or 2021 or 2020 or 2019 or 2018 or 2017 or 2016 or 2015 or 2014 or 2013 or 2012


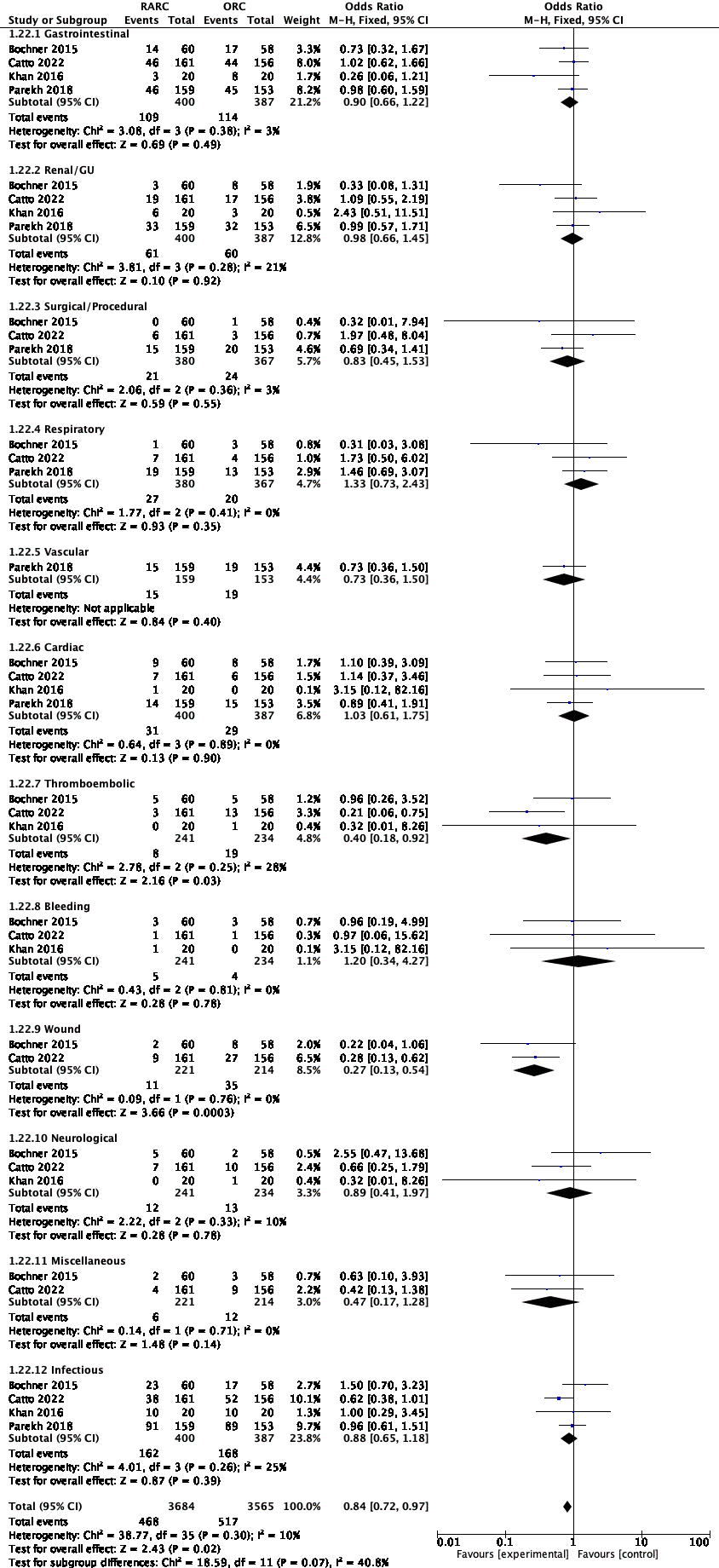
**Appendix B. Complication Type**


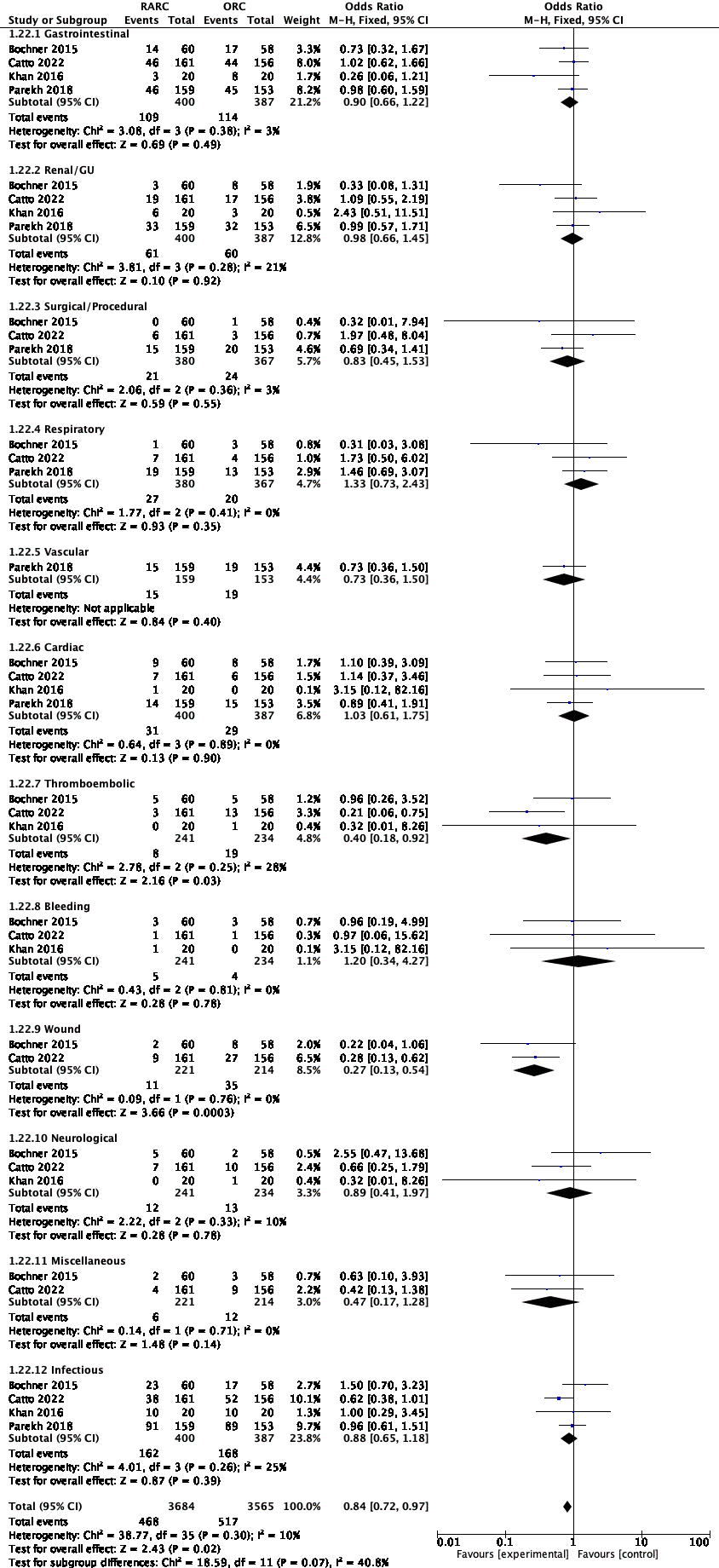


**Appendix C. Sensitivity Analyses**


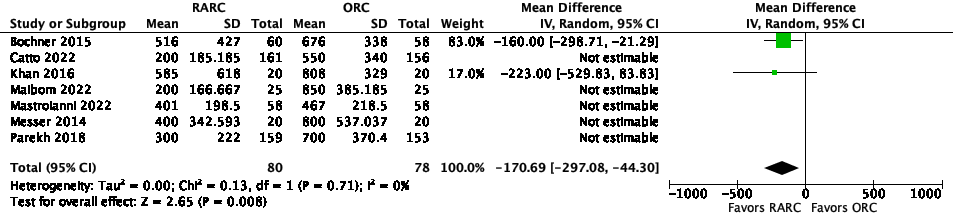


Sensitivity Analysis for Estimated Intraoperative Blood Loss


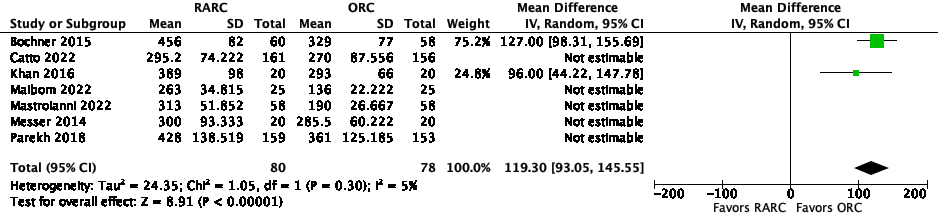


Sensitivity Analysis for Operative Time


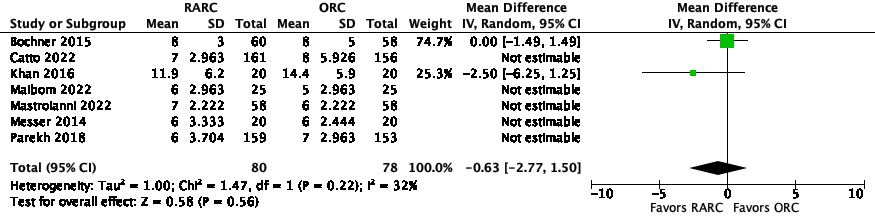


**Appendix D: Forest Plots of Study Outcomes**


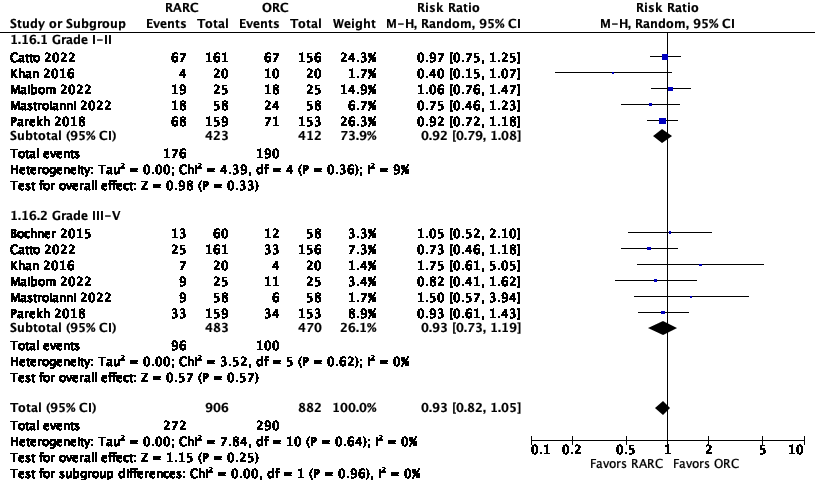


Forest plot of comparison: 1 Open vs Robot-Assisted Radical Cystectomy, outcome: 1.16 90d Complications


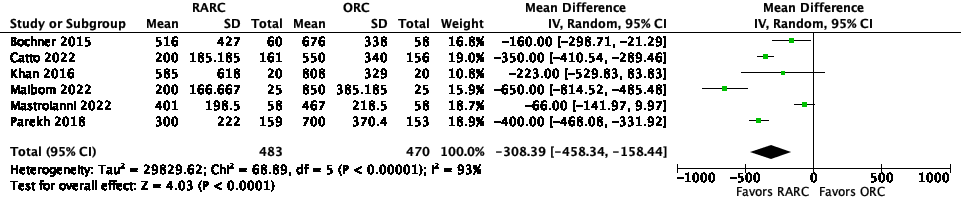


Forest plot of comparison: 1 Open vs Robot-Assisted Radical Cystectomy, outcome: 1.11 Intraoperative Blood Loss (EBL).


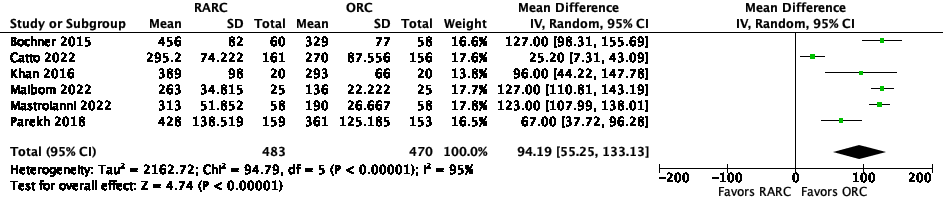


Forest plot of comparison: 1 Open vs Robot-Assisted Radical Cystectomy, outcome: 1.8 Operative Time.


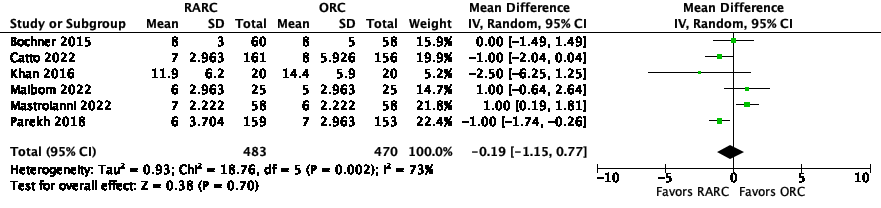


Forest plot of comparison: 1 Open vs Robot-Assisted Radical Cystectomy, outcome: 1.14 Length of Stay.


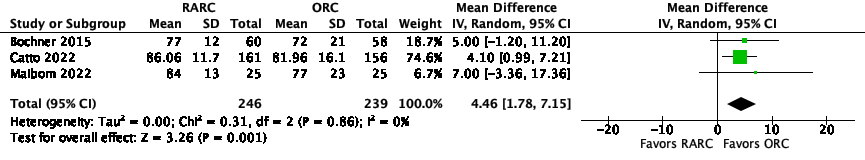


Forest plot of comparison: 1 Open vs Robot-Assisted Radical Cystectomy, outcome: 1.2 QoL EORTC QLQ-C30 (3 months).


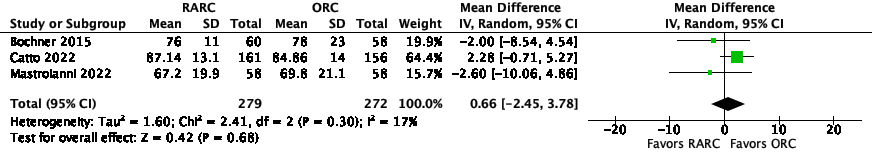


Forest plot of comparison: 1 Open vs Robot-Assisted Radical Cystectomy, outcome: 1.3 QoL EORTC QLQ-C30 (6 months).


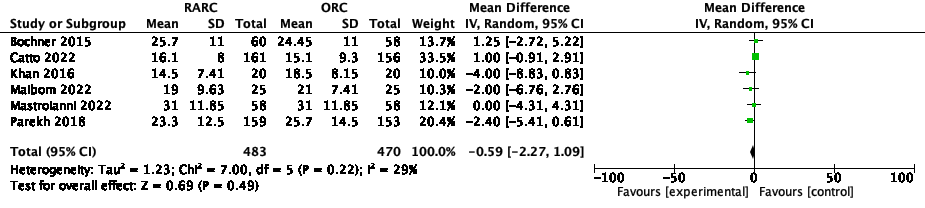


Forest plot of comparison: 1 Open vs Robot-Assisted Radical Cystectomy, outcome: 1.24 Lymph Node Yield.


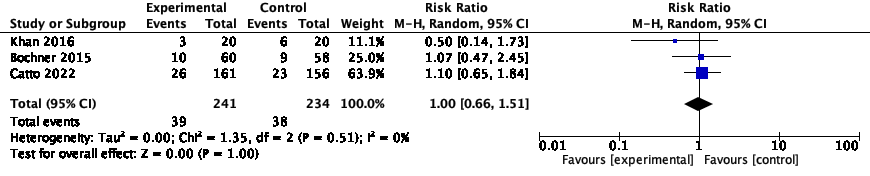


Forest plot of comparison: 1 Open vs Robot-Assisted Radical Cystectomy, outcome: 1.25 Lymph Nodes pos patients.


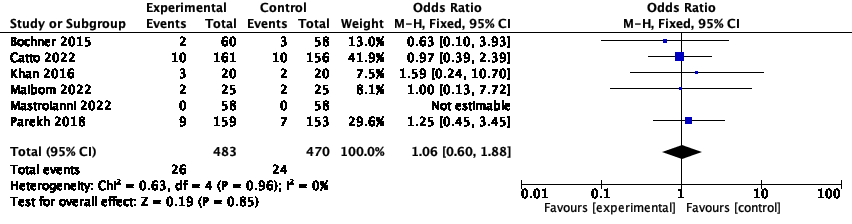


Forest plot of comparison: 1 Open vs Robot-Assisted Radical Cystectomy, outcome: 1.26 Positive
